# Supplementary material for: A Clinical Study on Urinary Clusterin and Cystatin B in Dogs with Spontaneous Acute Kidney Injury
Source: Vet Sci. 2024 May 2;11(5):200. doi: 10.3390/vetsci11050200 (PMC11125966; doi:10.3390/vetsci11050200)
Supplement: Supplementary file 1 [file vetsci-11-00200-s001.zip › vetsci-2941764-supplementary.pdf]

Figure S1. UClust/uCr and uCysB/uCr in dogs with AKI and healthy control dogs at inclusion

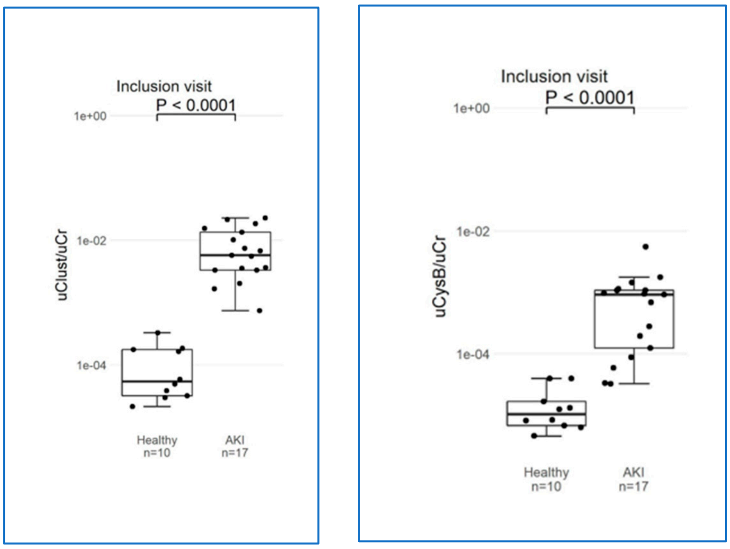

(a) (b)

Box-and-whisker plots of urinary clusterin/ urinary creatinine (uClust/uCr) (a) and urinary cystatin B/urinary creatinine (uCysB/uCr) (b) in dogs with acute kidney injury (AKI) and healthy control dogs (Healthy) at inclusion visit. Inclusion visit: <24 h from arrival at hospital (dogs with AKI) or study inclusion (healthy control dogs). *P*- values are obtained from Mann Whitney U- tests.

Figure S2. UClust/uCr and uCysB/uCr by initial AKI grade

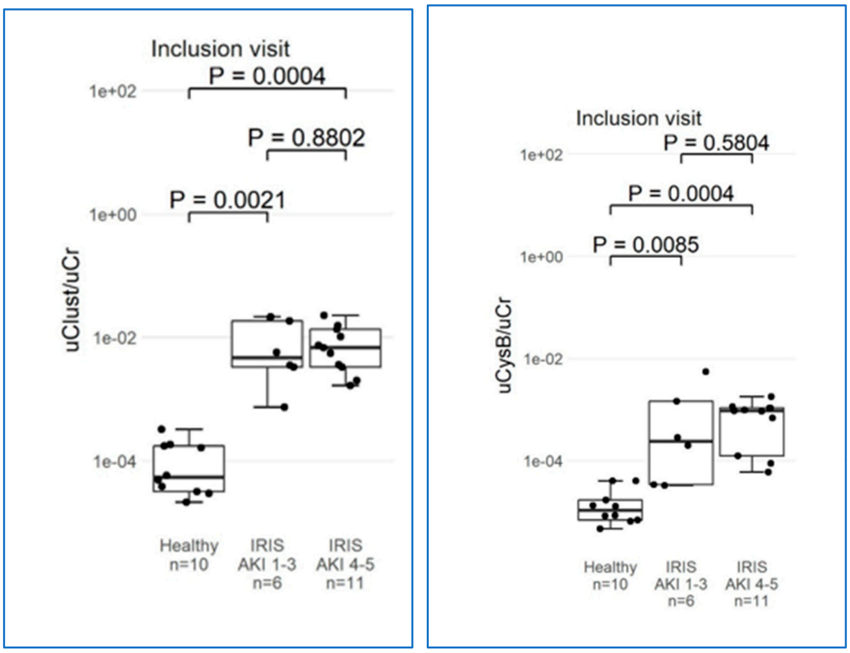

(a) (b)

Box-and-whisker plots of urinary clusterin/ urinary creatinine (uClust/uCr) (a) and urinary cystatin B/urinary creatinine (uCysB/uCr) (b) by initial acute kidney injury (AKI) grade. AKI grades are based on International Renal Interest Society (IRIS) AKI grading system on inclusion visit. Inclusion visit: <24 h from arrival at hospital (dogs with AKI) or study inclusion (healthy control dogs).

Figure S3. Graphs with biomarker levels (uClust, uCysB, crea) in dogs throughout the study

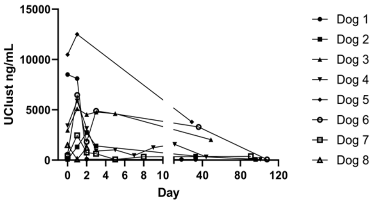

(a)

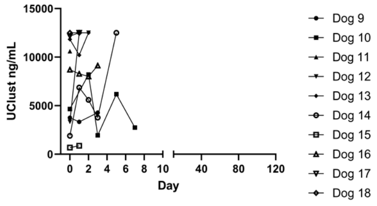

(b)

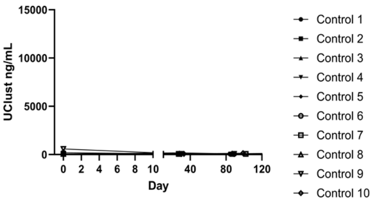

(c)

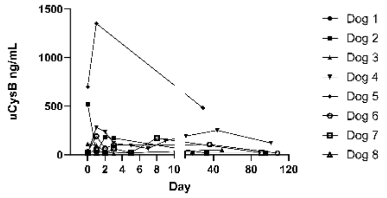

(d)

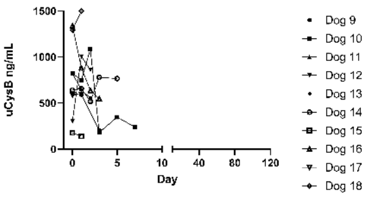

(e)

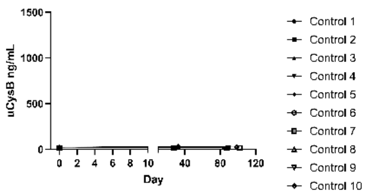

(f)

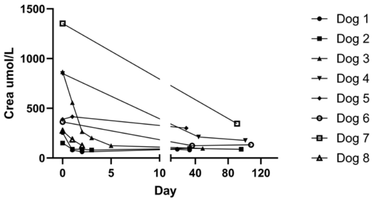

(g)

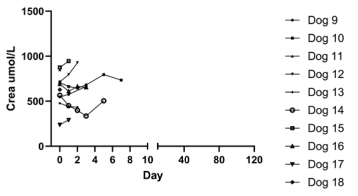

(h)

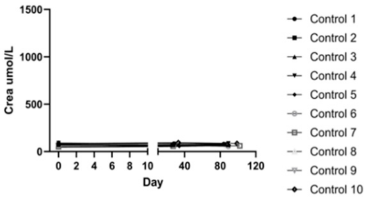

(i)

Graphs with biomarker levels (urinary clusterin, UClust; urinary cystatin B, UCysB; serum creatinine, Crea) in dogs. Graphs (a), (d) and (g) are showing dogs surviving one month or more from hospital discharge and examined at control visits (n=8). Graphs (b), (e) and (h) are showing dogs with AKI surviving less than one month from study inclusion (n=10). Graphs (c), (f) and (i) are showing healthy control dogs (n=10) during their time in the study (three months). In dogs treated with hemodialysis, only creatinine levels before treatment and from control visits are included in the graphs.
